# Supplementary material for: Aldo-Keto Reductase 1C3 Mediates Chemotherapy Resistance in Esophageal Adenocarcinoma via ROS Detoxification
Source: Cancers (Basel). 2021 May 16;13(10):2403. doi: 10.3390/cancers13102403 (PMC8156851; doi:10.3390/cancers13102403)
Supplement: Supplementary file 1 [file cancers-13-02403-s001.zip › cancers-1169193-supplementary.pdf]

Supplementary Materials: Aldo-Keto Reductase 1C3 Mediates Chemotherapy Resistance in Esophageal Adenocarcinoma via ROS Detoxification

Chenghui Zhou, Zhefang Wang, Jiahui Li, Xiaolin Wu, Ningbo Fan, Dai Li, Fanyu Liu, Patrick S. Plum, Sascha Hoppe, Axel M. Hillmer, Alexandar Quaas, Florian Gebauer, Seung-Hun Chon, Christiane J. Bruns and Yue Zhao

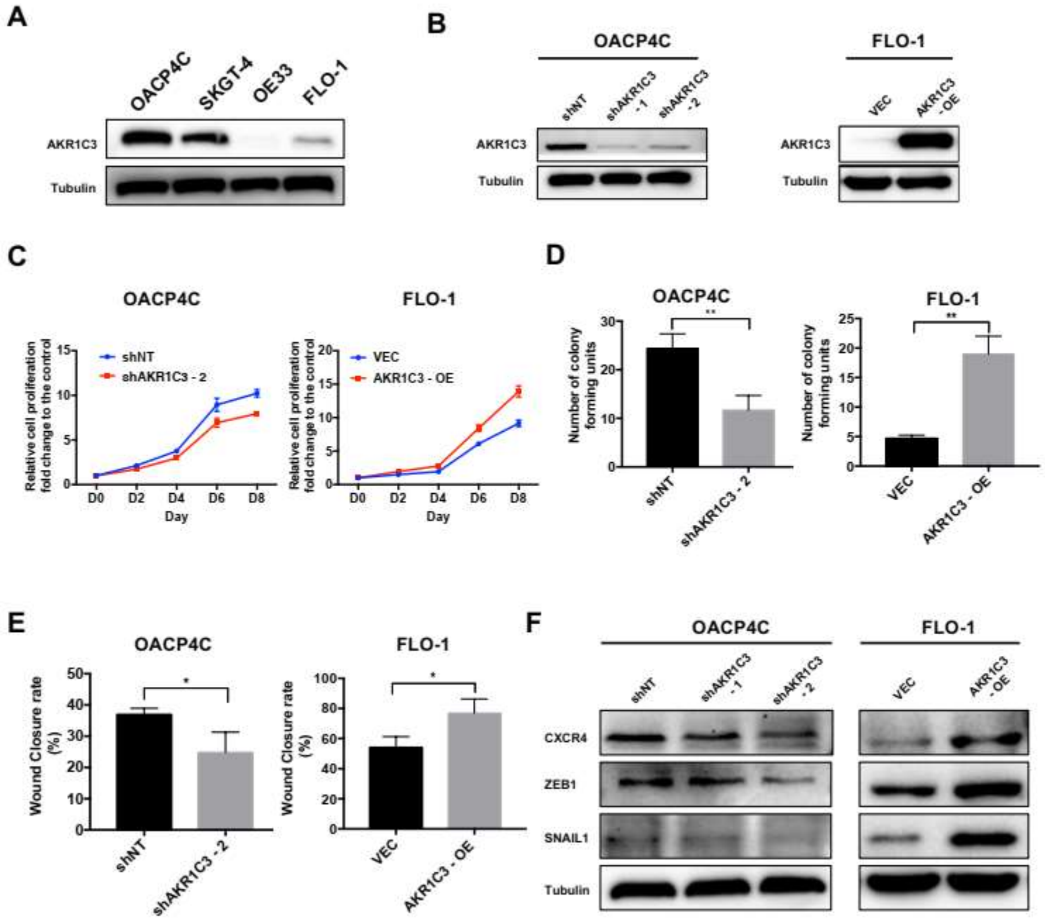

Figure S1. AKR1C3 promotes proliferation and colony formation and migration of EAC cells. (**A**) Relative expression level of endogenous AKR1C3 in 4 human EAC cell lines, SKGT-4, OACP4C, OE33 and FLO-1. (**B**) Validation of stable knockdown of AKR1C3 in OACP4C cells and overexpression of AKR1C3 in FLO-1 cells. (**C**) Proliferation rates, (**D**) colony forming capacity and (**E**) migration capacity of OACP4C shAKR1C3-2, FLO-1 AKR1C3 and their control cells were examined. (**F**) Western-blot results showed that the expression level of CXCR4, ZEB1 and SNAIL1 were downregulated when AKR1C3 was knocked down in OACP4C cells, but were upregulated when AKR1C3 was overexpressed in FLO-1 cells. Data are presented as mean ± SD (n = 3). * $p < 0.05$, ** $p < 0.01$, *** $p < 0.001$.

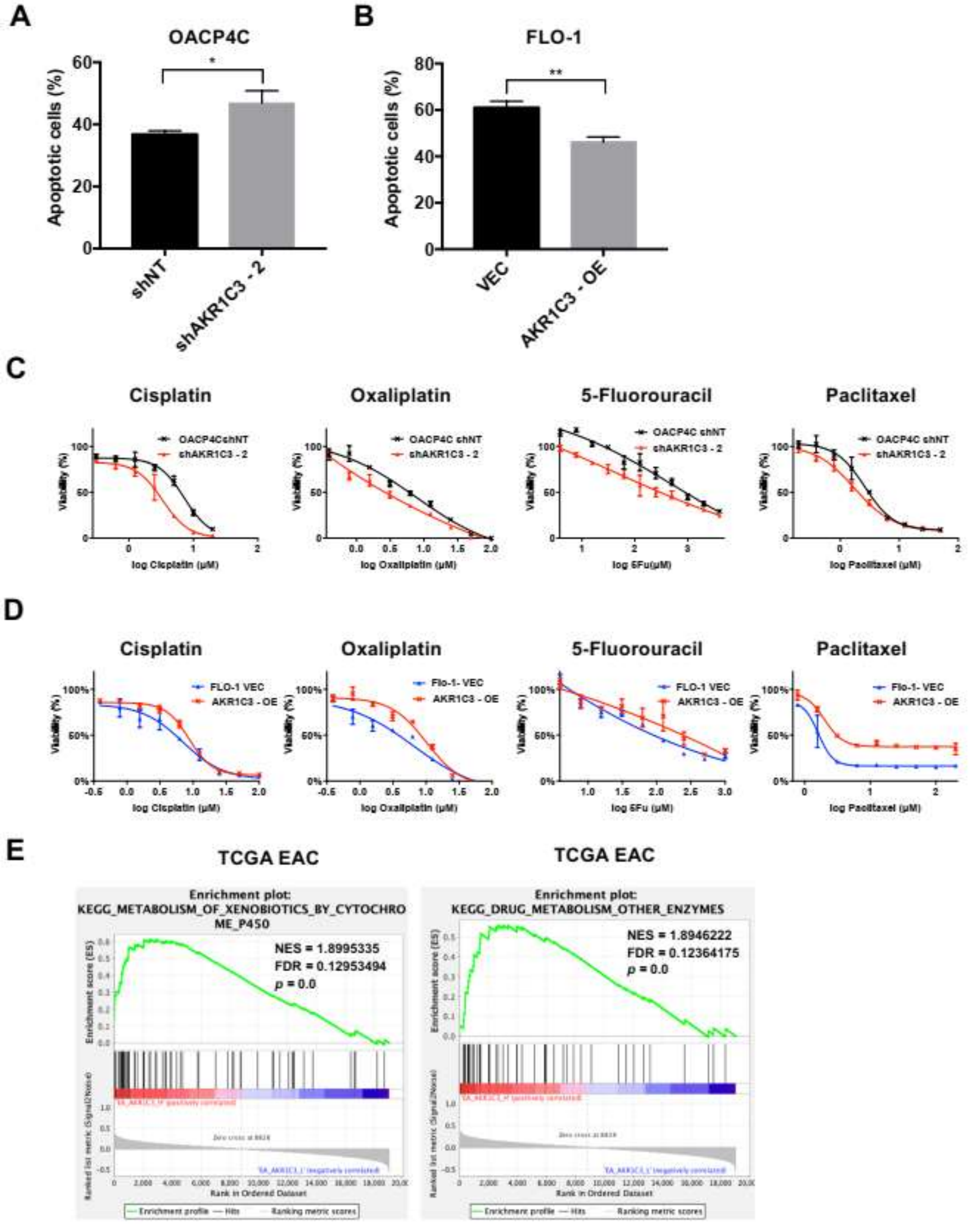

Scheme S2. AKR1C3 renders chemotherapy resistance in EAC cells. (**A**–**B**) Cells were treated with cisplatin (5 μM for OACP4C and 15 μM for FLO-1) for indicated time, and apoptosis was determined by flow cytometry analysis of Annexin V/DAPI staining. (**C**–**D**) Cell viability assay was used to determine cell viability after cisplatin, Oxaliplatin, 5-FU and paclitaxel treatment for 48 h in both OACP4C and FLO-1 cells. (**E**) GSEA analysis from TCGA dataset showed drug metabolism related enzymes is enriched in AKR1C3-high group. Bar graphs are presented as mean ± SD of three independent experiments. * $p < 0.05$, ** $p < 0.01$, *** $p < 0.001$, ns: non-significant, $p > 0.05$.

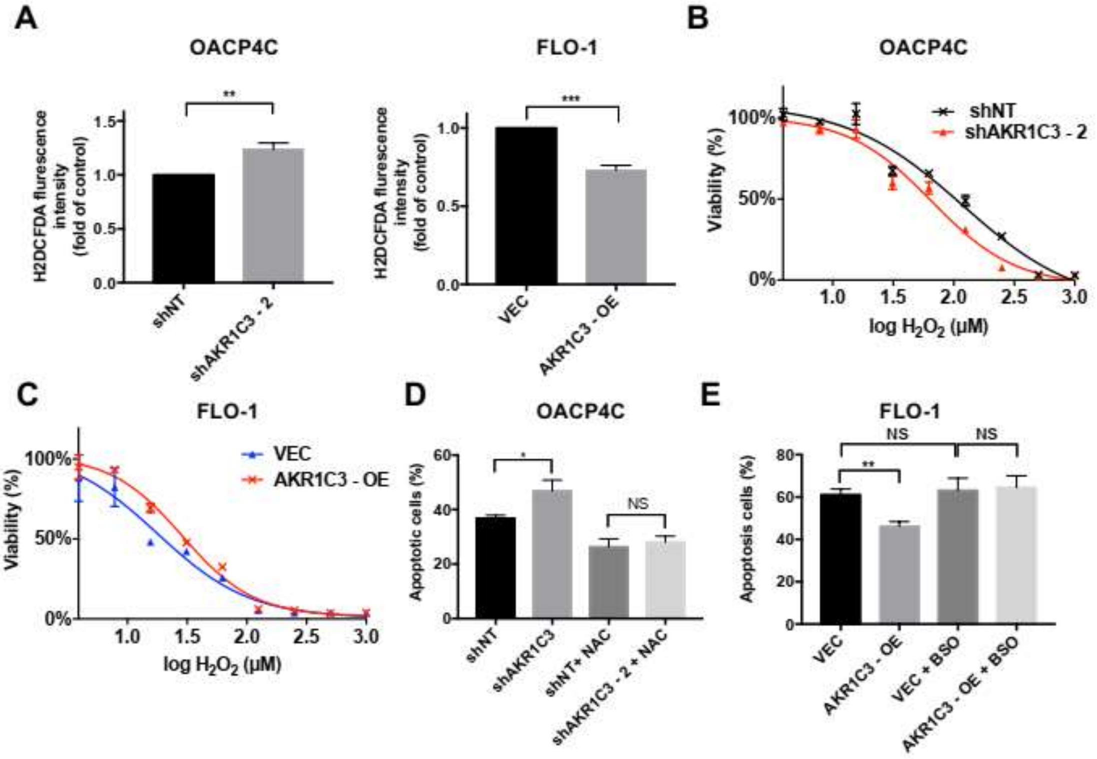

Figure S3. AKR1C3 mediates chemo-resistance through regulating redox-homeostasis. (**A**) Intracellular ROS production was measured by flow cytometry using the indicator dye H2DCFDA. Bar graphs are presented as mean ± SD of three independent experiments. (**B**–**C**) Cell viability assay was used to determine cell viability after Hydrogen Peroxide treatment for 48 h in OACP4C and FLO-1 respectively. (**D**) OACP4C cells were incubated with 4mM NAC and 10μM cisplatin for 48 h followed by measurement of apoptosis. (**E**) FLO-1 cells were incubated with 50 μM BSO and 15μM cisplatin for 48 h followed by measurement of apoptosis.

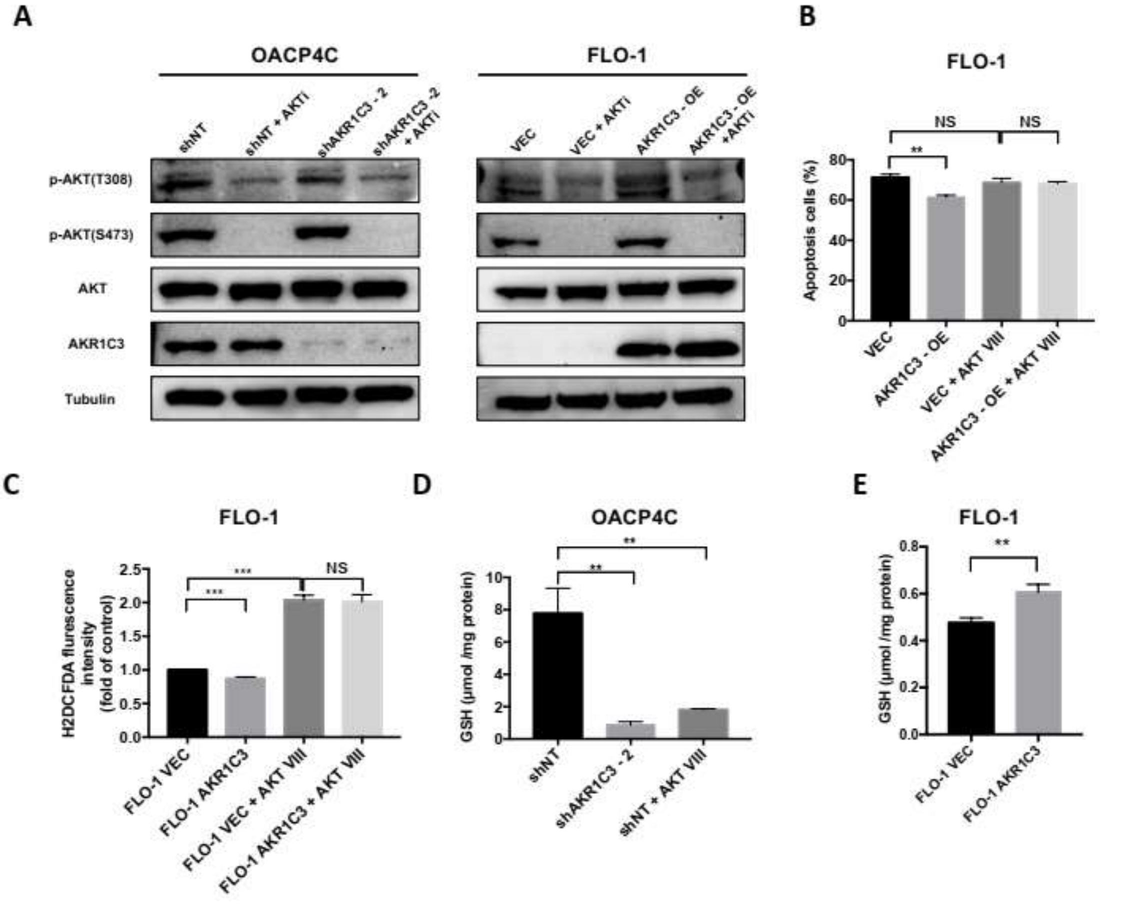

Figure S4. AKT phosphorylation is regulated by AKR1C3 and is responsible for ROS alleviation in EAC. (**A**) Western blot showed that the phosphorylation level of AKT was downregulated in OACP4C shAKR1C3 cells and upregulated in FLO-1 AKR1C3 overexpressing cells; and the phosphorylation level of AKT were lower in cells treated with 20 μM AKT inhibitor VIII than in cells without inhibitor, but total AKT protein level did not differ among groups. (**B**) FLO-1 cells were incubated with 20 μM AKT inhibitor VIII and 15 μM cisplatin for 48 h followed by measurement of apoptosis. (**C**) FLO-1 cells were incubated with 20 μM AKT inhibitor VIII for 24 h followed by measurement of ROS. (**D–E**) Data indicate glutathione concentration normalized to total protein content were decreased in OACP4C shAKR1C3 -2 cells and increased in FLO-1 AKR1C3 overexpressing cells, while glutathione concentration were lower in cells treated with 20 μM AKT inhibitor VIII than in cells without inhibitor. Data are shown as mean ± SD of triplicate samples. * $p < 0.05$, ** $p < 0.01$, *** $p < 0.001$, NS: non-significant, $p > 0.05$.

Table S1. Primers for qRT-PCR.

Gene	Sequence(5′-3′)
GAPDH-for	GAAGGTGAAGGTCGGAGTC
GAPDH-rev	GAAGATGGTGATGGGATTTC
hAKR1C3-for	GTCATCCGTATTTCAACCGGAG
hAKR1C3-rev	CCACCCATCGTTTGTCTCGTT
hNRF2-for	TTCCCGGTCACATCGAGAG
hNRF2-rev	TCCTGTTGCATACCGTCTAAATC
